# Radiosynthesis of [^11^C]Ibrutinib *via* Pd-Mediated [^11^C]CO Carbonylation: Preliminary PET Imaging in Experimental Autoimmune Encephalomyelitis Mice

**DOI:** 10.3389/fnume.2021.772289

**Published:** 2021-11-12

**Authors:** Anton Lindberg, Amanda J. Boyle, Junchao Tong, Michael B. Harkness, Armando Garcia, Tritin Tran, Dongxu Zhai, Fang Liu, David J. Donnelly, Neil Vasdev

**Affiliations:** ^1^Azrieli Centre for Neuro-Radiochemistry, Centre for Addiction and Mental Health, Toronto, ON, Canada; ^2^Brain Health Imaging Centre, Centre for Addiction and Mental Health, Campbell Family Mental Health Research Institute, Toronto, ON, Canada; ^3^Bristol-Myers Squibb Research and Development, New York, NJ, United States; ^4^Department of Psychiatry, University of Toronto, Toronto, ON, Canada; ^5^Department of Physiology, University of Toronto, Toronto, ON, Canada

**Keywords:** ibrutinib, multiple sclerosis, positron emission tomography, carbon-11, carbon monoxide

## Abstract

Ibrutinib is a first-generation Bruton's tyrosine kinase (BTK) inhibitor that has shown efficacy in autoimmune diseases and has consequently been developed as a positron emission tomography (PET) radiotracer. Herein, we report the automated radiosynthesis of [^11^C]ibrutinib through ^11^C-carbonylation of the acrylamide functional group, by reaction of the secondary amine precursor with [^11^C]CO, iodoethylene, and palladium–NiXantphos. [^11^C]Ibrutinib was reliably formulated in radiochemical yields of 5.4% ± 2.5% (non-decay corrected; *n* = 9, relative to starting [^11^C]CO_2_), radiochemical purity >99%, and molar activity of 58.8 ± 30.8 GBq/μmol (1.55 ± 0.83 Ci/μmol). Preliminary PET/magnetic resonance imaging with [^11^C]ibrutinib in experimental autoimmune encephalomyelitis (EAE) mice showed a 49% higher radioactivity accumulation in the spinal cord of mice with EAE scores of 2.5 vs. sham mice.

## Introduction

Bruton's tyrosine kinase (BTK) is critical for the maturation of B cells and in regulating hematopoietic cell circulation (1, 2). BTK inhibitors have been developed to treat B-cell malignancies and autoimmune diseases including multiple sclerosis (MS) (3, 4), where it is postulated that inhibiting BTK blocks the maturation of B cells (5–9). Ibrutinib is a first-generation BTK inhibitor used as a chemotherapeutic agent to treat B-cell cancers (10). Although ibrutinib has not been studied clinically as a BTK inhibitor in MS, it has shown efficacy in other autoimmune diseases, such as arthritis, lupus, and recently in treatment of COVID-19 (SARS-CoV-2) (11–14). The versatility of BTK inhibitors, such as ibrutinib, in treating various B-cell-related diseases has led to their development as radiotracers for positron emission tomography (PET) (15, 16).

**Figure 1:**
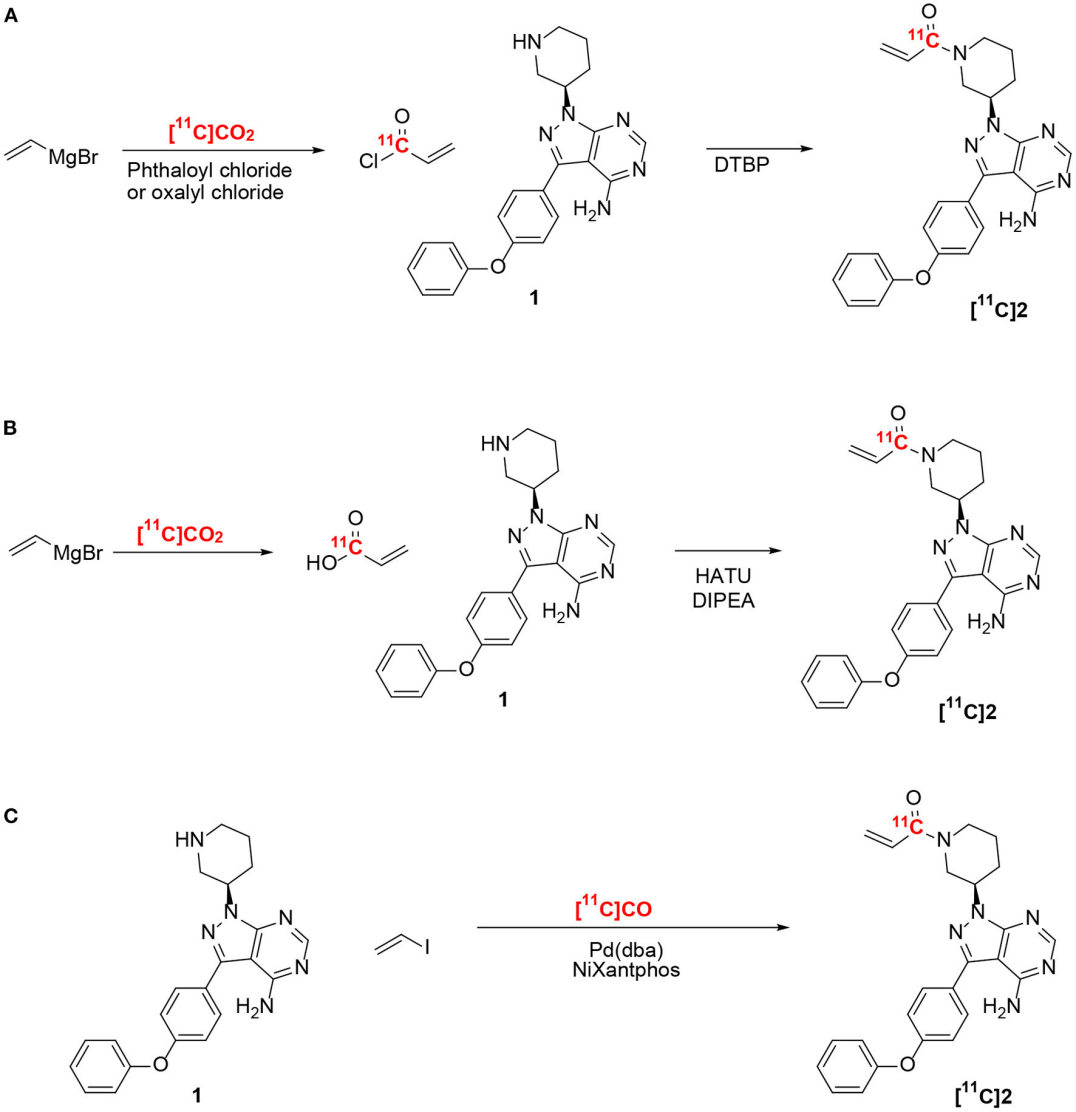
Radiosynthesis of [^11^C]ibrutinib ([^11^C]2). **(A,B)** Previously reported methods using ethylene magnesium bromide and [^11^C]CO_2_ (16). **(C)** This work.

Ibrutinib (**2**) has been labeled at the acrylamide functionality with carbon-11 (^11^C) using [^11^C]CO_2_ (Figure 1) (16). However, the method resulted in low radiochemical yields (RCYs) (<5%, non-decay corrected) due to side reactions caused by the Grignard reagents, which also affected the chemical purity and radiochemical purity (RCP). Although the chemical purity and RCP were improved by using carrier-added CO_2_, this resulted in low molar activity (*A*_m_), 2.0 GBq/μmol (~55 mCi/μmol). The above-mentioned limitations (low RCP, chemical purity, *A*_m_, and RCY) have restricted the widespread use of [^11^C]ibrutinib for PET imaging. Hence, a reliable radiosynthesis of [^11^C]ibrutinib is needed with improved RCY, RCP, and *A*_m_, ideally by avoiding Grignard reagents and carrier-added CO_2_.

Radiolabeling of carbonyls such as acrylamides has been extensively reported using [^11^C]CO_2_ (17, 18). Recent developments in converting cyclotron-produced [^11^C]CO_2_ to [^11^C]CO have provided access to new radiosynthesis pathways for ^11^C-carbonylation (19–23). We recently reported the labeling of the acrylamide moiety of [^11^C]tolebrutinib by ^11^C-carbonylation *via* [^11^C]CO, iodoethylene, and the secondary amine precursor using a NiXantphos-mediated protocol under atmospheric pressure using no-carrier-added [^11^C]CO (Figure 1) (15). In the present study, we have adapted this radiosynthesis to label [^11^C]ibrutinib *via* ^11^C-carbonylation of 3-(4-phenoxyphenyl)-1-(piperidin-3-yl)-2,3-dihydro-1*H*-pyrazolo[3,4-*d*]pyrimidin-4-amine **(1**), and we report a preliminary PET/magnetic resonance imaging (MRI) study of the spinal cord in experimental autoimmune encephalomyelitis (EAE) mouse models.

## Results and Discussion

The efficacy of ibrutinib in autoimmune diseases, such as arthritis, lupus, and COVID-19 (SARS-CoV-2), makes [^11^C]ibrutinib an ideal BTK PET radiotracer. The *N*-acrylamide moiety of ibrutinib also lends this structure well for [^11^C]CO carbonylation utilizing a new Pd-mediated methodology that we recently reported for the radiolabeling of another BTK inhibitor, [^11^C]tolebrutinib (15). Our [^11^C]CO carbonylation of [^11^C]tolebrutinib has advantages over the recently reported [^11^C]CO_2_ carbonylation of [^11^C]ibrutinib including higher RCYs and does not require carrier-added CO_2_, thereby also improving the *A*_m_ of the radiotracer (Table 1) (15, 16). As ibrutinib is well tolerated as a pharmaceutical, and as the authentic standard and amine precursor for [^11^C]CO carbonylation are commercially available, [^11^C]ibrutinib could be rapidly validated on an automated synthesis unit for human use.

**Table 1:** Radiochemical syntheses of [^11^C]ibrutinib.

**Radiochemical method**	**RCY (non-decay corrected)**	**RCP**	***A*_**m**_**
[^11^C]CO_2_ carbonylation (carrier added)	5%	99%	2.0 GBq/μmol
[^11^C]CO_2_ carbonylation (no carrier added)	2–5%	90%	22–44 GBq/μmol
[^11^C]CO carbonylation This work	>6%	99%	59 GBq/μmol

*RCY, radiochemical yield; RCP, radiochemical purity*.

### Radiosynthesis of [^11^C]Ibrutinib

**Figure 2:**
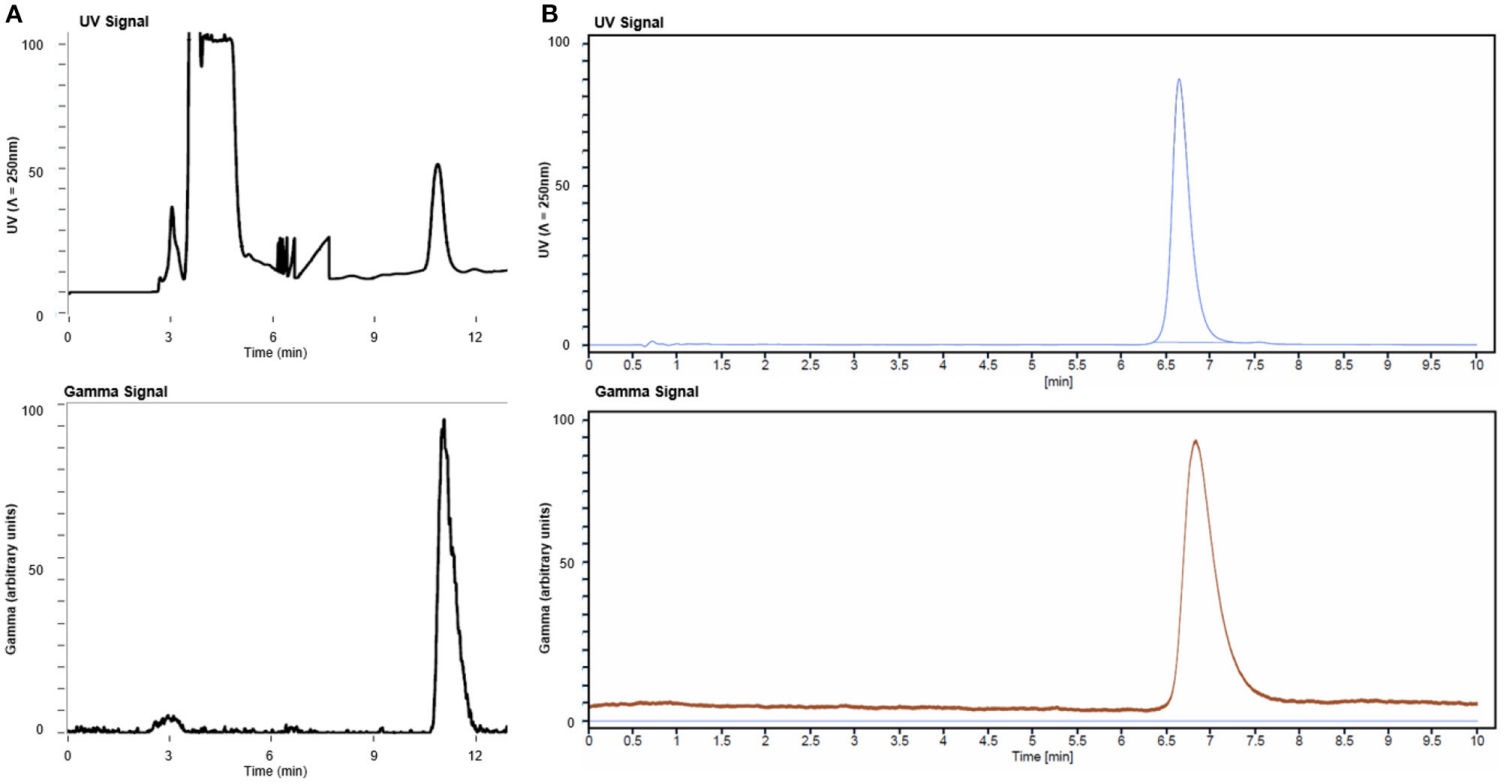
**(A)** Semi-preparative high-performance liquid chromatography (HPLC) chromatogram from production of [^11^C]ibrutinib and **(B)** analytical HPLC chromatograms of [^11^C]ibrutinib co-injected with ibrutinib standard.

The radiosynthesis of [^11^C]ibrutinib was fully automated using a commercial TracerMaker™ ^11^C synthesis platform. The method and setup of the automated synthesis platform were adapted with only minor changes from our previously published method for [^11^C]tolebrutinib (15). Commercially available anhydrous tetrahydrofuran (THF) solvent was used, eliminating the need for freshly distilled THF, and the semi-preparative high-performance liquid chromatography (HPLC) employed a flow rate of 6.0 ml/min. [^11^C]Ibrutinib was produced in 45–50 min overall synthesis time from end of bombardment (EOB) with a retention time of 12–13 min (Figure 2A). [^11^C]Ibrutinib was synthesized in 5.4 ± 2.5% (*n* = 9) RCY (non-decay corrected) relative to [^11^C]CO_2_, with RCP >99%, as confirmed by co-injection of authentic standard (Figure 2B), and *A*_m_ of 58.8 ± 30.8 GBq/μmol (1.55 ± 0.83 Ci/μmol) as measured by HPLC. The chromatogram from the semi-preparative HPLC purification shows only a minor radioactive by-product eluting at 3 min (Figure 2A). No radioactive by-products were seen in the analytical HPLC chromatograms (Figure 2B), indicating that the [^11^C]CO carbonylation reaction under these conditions is highly selective for the secondary *N*-piperidinyl moiety over the primary pyrimidin-4-amine.

### Preliminary PET/MR Imaging and Biodistribution in an Experimental Autoimmune Encephalomyelitis Mouse Model

**Figure 3:**
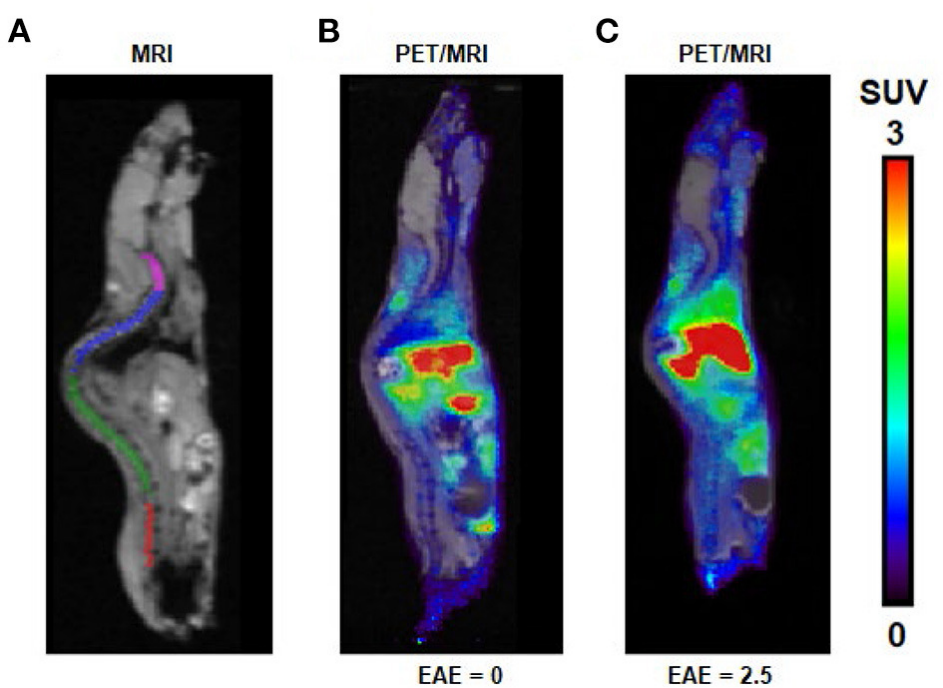
PET/MR images following intravenous (i.v.) injection of [^11^C]ibrutinib in experimental autoimmune encephalomyelitis (EAE) mouse models. **(A)** MRI indicating regions of interest (ROIs) of cervical (magenta), thoracic (blue), lumbar (green), and sacral (red) regions of the spinal cord. Representative sagittal PET/MR images (summed 0–60 min) following i.v. injection of [^11^C]ibrutinib in mice with EAE scores of **(B)** 0.0 and **(C)** 2.5.

**Figure 4:**
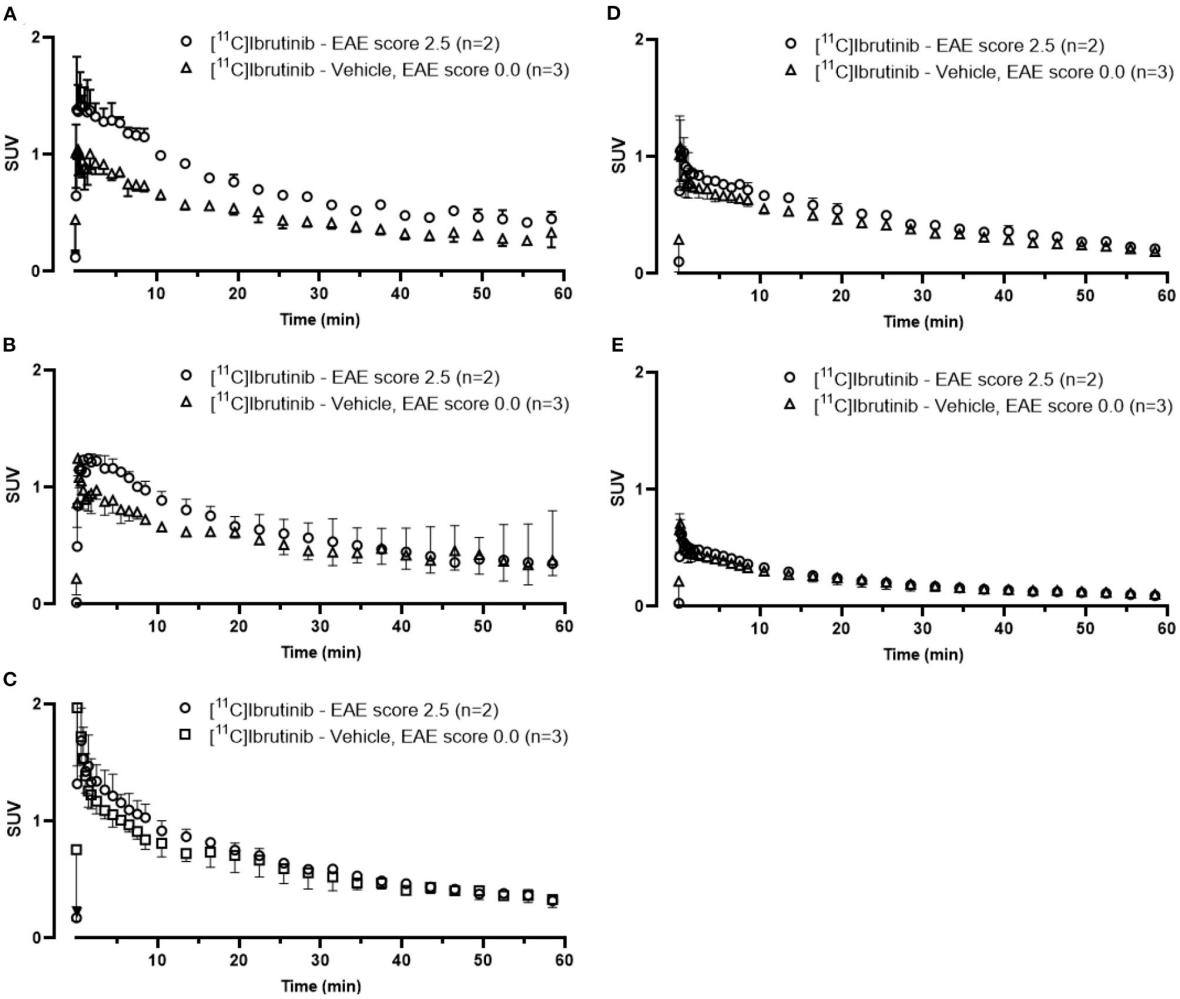
Time activity curves (TACs) from 0 to 60 min associated with PET images [^11^C]ibrutinib in mice with experimental autoimmune encephalomyelitis (EAE) scores 0.0 and 2.5. TACs were extracted from regions of interest (ROIs) in the **(A)** sacral, **(B)** lumbar, **(C)** thoracic, and **(D)** cervical regions of the spinal cord and in the **(E)** whole brain.

We evaluated the utility of [^11^C]ibrutinib in EAE mouse models of MS using female C57BL/6 mice (EAE score = 2.5) as compared with sham-treated animals (EAE score = 0.0). Regions of interest (ROIs) of the spinal cord were selected from the MRI (Figure 3A). Figures 3B,C show representative static PET/MR images with [^11^C]ibrutinib (0–60 min average image) in the EAE mice compared with the sham group, respectively. As demonstrated in the time activity curves (TACs) shown in Figure 4A, a 49% higher accumulation of radioactivity was measured in the sacral section of the spinal cord of the EAE mice (EAE score = 2.5) as compared with the sham at 5.5 min post-injection (p.i.), 1.27 ± 0.49 and 0.85 ± 0.04, respectively (*p* = 0.0409). Radioactivity accumulation decreased gradually but remained higher in EAE mice compared with sham mice until 40.5 min p.i., 0.48 ± 0.03 and 0.32 ± 0.05, respectively (*p* = 0.0336). Figure 4B shows that a 32% higher accumulation of radioactivity was measured in the lumbar region of EAE mice (EAE score = 2.5) as compared with the sham mice at 5.5 min p.i., 1.13 ± 0.37 and 0.80 ± 0.12, respectively (*p* = 0.0359). Radioactivity accumulation in the lumbar region of EAE mice cleared faster than in the sacral region of EAE mice, remaining higher than sham mice until 13.5 min p.i., 0.81 ± 0.09 and 0.62 ± 0.002, respectively (*p* = 0.03). Figures 4C,D show the thoracic and cervical sections of the spinal cord, respectively, for which there was no statistical difference from 0 to 60 min p.i. between the EAE mice and the sham mice. In accordance with previously published PET imaging results (16), whole-brain uptake was low, with SUV <0.5 from 5 to 60 min (Figure 4E). Biodistribution of the spinal cord (lumbar and thoracic regions combined) in mice sacrificed 60 min p.i. with [^11^C]ibrutinib was 0.17% ID/g and 0.09% ID/g, in EAE mice and sham, respectively. The sacral region of the spinal is too small in this mouse model to be quickly and reliably extracted. Despite this time point being past the peak of radioactivity accumulation of 5 min, as well as excluding the sacral region, the results indicate a trend toward higher radioactivity accumulation in the EAE spinal cord compared with sham spinal cord. A limitation of our PET imaging studies is the low power of our animal studies (*n* = 2–3), and future work would involve increasing the sample size.

## Conclusions

A fully automated and reliable radiosynthesis of [^11^C]ibrutinib using [^11^C]CO has been developed. [^11^C]Ibrutinib was synthesized in RCYs (5.4 ± 2.5%, non-decay corrected), RCP (>99%), and *A*_m_ (58.8 ± 32.8 GBq/μmol). Preliminary PET imaging studies in EAE mouse models show a trend toward increased radioactivity accumulation in the spinal cord of mice with higher EAE scores. Further studies that demonstrate the specificity of [^11^C]ibrutinib for imaging BTK *in vivo* are warranted, and the investigation of [^11^C]ibrutinib as a PET radiotracer in other B-cell disease models is possible.

## Materials and Methods

### Chemistry

Reference standards of ibrutinib were acquired from Toronto Research Chemicals (Toronto, ON, Canada). 3-(4-Phenoxyphenyl)-1-(piperidin-3-yl)-2,3-dihydro-1*H*-pyrazolo[3,4-*d*]pyrimidin-4-amine (**1**) was provided by Bristol Myer-Squibb Pharmaceuticals (Princeton, NJ, USA). Iodoethylene was acquired from Oakwood Chemicals (Estill, SC, USA). All other chemicals were acquired from Sigma-Aldrich (USA or Canada). No-carrier-added [^11^C]CO_2_ was produced on a MC-17 Cyclotron (Scanditronix, Vislanda, Sweden) using the ^14^N(p,α)^11^C nuclear reaction in a pressurized gas target containing a nitrogen:oxygen mixture (99.5:0.5) The radiosynthesis was performed using a TracerMaker™ carbon-11 synthesis platform (Scansys Laboratorieteknik, Værløse, Denmark). Analytical HPLC was performed on a 1260 infinity II HPLC system (Agilent, Santa Clara, CA, USA) with a M177 γ-radiation detector (Ludlum Instruments, Sweetwater, TX, USA) connected in series after the UV detector.

### Radiosynthesis of [^11^C]Ibrutinib

The reaction mixture was prepared in advance by weighing bis(dibenzylideneacetone)palladium(0) (2 mg, 3.5 μmol) and NiXantphos (2 mg, 3.5 μmol) in a capped vial under argon atmosphere, 20 min before EOB iodoethylene in THF was added (0.25% v/v, 600 μL) and vortexed for 2 min. Five minute before EOB, the iodoethylene/palladium mixture was added to a reaction vessel pre-charged with 3-(4-phenoxyphenyl)-1-(piperidin-3-yl)-2,3-dihydro-1*H*-pyrazolo [3,4-*d*]pyrimidin-4-amine (3 mg, 7.5 μmol) under an argon atmosphere using a balloon.

Cyclotron produced non-carrier-added [^11^C]CO_2_ was first trapped on a Hayesep D column (700 mg) cooled to −180°C using liquid nitrogen on a TracerMaker™ synthesis platform. The Hayesep D column was first slowly heated to ambient temperature and further reduced online to [^11^C]CO over heated (850°C) molybdenum powder (350 μm, GoodFellow, Huntingdon, UK) and concentrated on silica gel trap immersed in liquid nitrogen. After completed entrapment, the trap was heated to ambient temperature to release [^11^C]CO and bubble through a closed reaction vessel (4 ml) containing the prepared reaction mixture. The reaction was then heated to 100°C, 3 min before being cooled to 50°C, and vented under helium flow (2 ml/min) for 1 min to evaporate excess THF. The mixture was redissolved in MeCN:H_2_O (30:70, 3 ml) before being injected onto a semi-preparative HPLC column (Gemini C18 10 μm, 250 × 10 mm, Phenomenex, Torrance, CA, USA). [^11^C]Ibrutinib was eluted with MeCN:ammonium acetate (45:55 v/v, 30 mM) at 6 ml/min (t_R_ = 11–13 min). Selected fraction was diluted with H_2_O (15 ml) and pushed through a solid-phase extraction (SPE) cartridge (SepPak tC18, Waters, Milford, MA, USA). The SPE cartridge was washed with water (10 ml) before [^11^C]ibrutinib was eluted with ethanol (1 ml) and formulated with saline (0.9%, 9 ml). The overall synthesis time from EOB was 45–49 min.

RCP and molar activity were determined by analytical HPLC (Alltima C18 5 μm, 250 × 4.5 mm, HiChrom, Leicestershire, UK). [^11^C]Ibrutinib was eluted with MeCN:ammonium formate (50:50 v/v, 50 mM) at 2 ml/min (t_R_ = 7 min). Identity was confirmed by co-injection with verified reference standard. *A*_m_ was calculated by comparison with a reference standard of known concentration (1.25 mg in 50 ml). RCY was calculated by comparing radioactivity at EOB to radioactivity of formulated product.

### Experimental Autoimmune Encephalomyelitis Mouse Models of Multiple Sclerosis

A group of two C57BL/6 mice (2-month-old) was immunized with 200 μg of myelin oligoglycoprotein (MOG35-55) (CSBio, Menlo Park, CA, USA), prepared in a 1:1 emulsion of complete Freund's adjuvant (Difco, Franklin Lakes, NJ, USA) to a concentration of 2 mg/ml injected subcutaneously in the lower back near the base of the tail. Within 2 h of MOG35-55 injection, mice received an intraperitoneal (i.p.) injection of 200 ng of pertussis toxin (List Biological Laboratories, Campbell, CA, USA) and again 24 h later. Mice were monitored daily for clinical signs of EAE, including body weight and standard EAE scoring (0, no paralysis; 1, limp tail; 2, limp tail with hind leg weakness; 3, limp tail with hind leg paralysis; 4, limp tail with complete hind leg paralysis and partial front leg paralysis; and 5, moribund or deceased). Mice did not progress past an EAE score of 2.5. A control sham group of mice was injected with saline as described above (*n* = 3). Animal studies were conducted under a protocol (#793) approved by the Animal Care Committee at the Centre for Additions and Mental Health, following Canadian Council on Animal Care guidelines. PET imaging was performed when mice reached an EAE score of 2.5, ~2 weeks post-induction, and the sham group with an EAE score of 0.0 was also imaged at ~2 weeks of saline injection.

### PET Acquisition Method

For small animal PET combined with MR imaging studies, mice were anesthetized by isoflurane in O_2_ (4%, 2 L/min induction; 1%−2%, 1 L/min maintenance) for lateral tail-vein catheterization and then transferred to a nanoScan™ PET/MRI 3T scanner (Mediso, Budapest, Hungary). Mice with an EAE score of 2.5 (*n* = 2) or 0.0 (*n* = 3) were injected through the tail-vein catheter with [^11^C]ibrutinib (5.61–9.27 MBq, 151–250 μCi, 5.20–11.81 nmol/kg, 24–74 GBq/μmol, 648–2,000 mCi/μmol). Anesthesia was maintained throughout PET/MR scanning, while body temperature and respiration parameters were monitored. A short gradient echo (GRE) scout MR was acquired for positioning the mouse in the PET field of view followed by a T1-weighted material map MR acquisition (GRE 3D, repetition time (TR) 25 ms, echo time (TE) 4.76 ms) for PET and MR co-registration and PET scatter and attenuation corrections. PET scans were initiated at the time of radioligand injection, and the list mode data were acquired for 60 min with an energy window of 400–600 keV. Following PET scanning, the animals were sacrificed by cervical dislocation, and the spinal cord was collected, weighed, and transferred to γ-counting tubes for biodistribution determination. Tissue radioactivity was measured with a γ-counter and expressed as %ID/g.

### PET Data Analyses

List mode data were sorted into 33, 3D (3 × 5, 3 × 15, 3 × 20, 7 × 60, and 17 × 180 s) true sinograms (ring difference 84). 3D sinograms were converted in 2D sinograms using Fourier rebinning (24) with corrections for detector geometry, efficiencies, attenuation, and scatter, prior to image reconstruction using a 2D-filtered backprojection (FBKP) with a Hann filter at a cutoff of 0.50 cm^−1^. A static image of the complete emission acquisition was reconstructed with the manufacturer's iterative 3D algorithm (six subsets, four iterations). The static iterative image was used for PET and MR co-registration and for presentation in figures. All data were corrected for dead-time and decay corrected to the start of acquisition. Dynamic FBKP images were used to extract TACs. Image analysis of the spinal cord (sacral, lumbar, thoracic, and cervical sections drawn manually) and brain (using the MR atlas of C57Bl/6J mice) (25) was performed using VivoQuant (4.0 patch1, Invicro LLC, Boston, MA, USA) and expressed as standardized uptake value (SUV), assuming tissue density of 1 g/ml.

### Statistical Analysis

Data are represented as the mean ± SD. Statistical comparisons were performed by an unpaired *t*-test (*p* < 0.05) with GraphPad Prism Version 8.3.1.

## Data Availability Statement

The raw data supporting the conclusions of this article will be made available by the authors, without undue reservation.

## Ethics Statement

The animal study was reviewed and approved by CAMH Animal Care Committee.

## Author Contributions

AL, AB, DD, and NV designed the research. AL, AB, JT, MH, AG, TT, and DZ performed the research. AL, AB, JT, FL, DD, and NV analyzed the data. AL, AB, and NV wrote the paper. All authors reviewed the manuscript.

## Dedication

This work is dedicated to the memory of TT, who passed away suddenly during the preparation of this manuscript. TT was a talented chemist who worked passionately on many PET ligand discovery projects. His enthusiasm for science will be missed within the field.

## Conflict of Interest

NV is a co-founder of MedChem Imaging, Inc. DD and TT are employed by Bristol-Myers Squibb Research and Development. The remaining authors declare that the research was conducted in the absence of any commercial or financial relationships that could be construed as a potential conflict of interest.

## Publisher's Note

All claims expressed in this article are solely those of the authors and do not necessarily represent those of their affiliated organizations, or those of the publisher, the editors and the reviewers. Any product that may be evaluated in this article, or claim that may be made by its manufacturer, is not guaranteed or endorsed by the publisher.
